# Supplementary material for: Mapping QTL Associated with Resistance to Avian Oncogenic Marek’s Disease Virus (MDV) Reveals Major Candidate Genes and Variants
Source: Genes (Basel). 2020 Aug 30;11(9):1019. doi: 10.3390/genes11091019 (PMC7564597; doi:10.3390/genes11091019)
Supplement: Supplementary file 1 [file genes-11-01019-s001.zip › S1 File.docx]

**QTLR 5 on Chr 1**

All 4 elements tested in this QTLR were significant in Lines WL2 and RIR1, and across lines (**Supplementary Table 8**). In Line WL3 the genes CSTA and C1S were significant, but not miRNA 6553 or the gene LAG3 between them. This patterns of P-value distribution suggest two independent quantitative elements in QTLR 5. Furthermore, the gene *PRSS3*, immediately upstream of *LAG3*, also genotyped in this study, gave indications of gene duplication and hence could not be genotyped, but could possibly be the causative QTG near *LAG3*.

**QTLR 7 on Chr 1**

Markers in the *ADAMTS5* gene were highly significant intermittently in Line WPR1 and across lines. The distribution of P-values suggests interdigitated haplotype blocks (Lipkin et al., 2013) in Lines WL3, WPR1, WPR2, WL4 and RIR1. The lack of within-line significant tests in miRNA 1738, located 1.6 MB downstream in the same QTLR, suggests that *ADAMTS5* or a nearby element is the putative QTG in this QTLR.

**QTLR 8 on Chr 1**

Significant to highly significant results were found in upstream markers and haplotypes of *SUPT20H* in the white egg Line WL2, in downstream markers in the brown egg Line RIR1, and across lines. Thus, here too possibly 2 different elements are responsible to the QTLR quantitative effect. The complex haplotype blocks indicated by the P-value pattern support this notion.

**QTLR 10 on Chr 1**

Fifteen SNPs located in the exons of *FLT3* were used to validate this QTLR. All SNPs were non-synonymous and involved amino acid (AA) substitution (**Supplementary Tables 1 and 7**). Significant association was found in all three brown egg lines WPR1, WPR2 and RIR1, and in the white egg line WL4. However, a complex pattern of P-values makes it difficult to locate the precise putative causative element in this gene/region.

**QTLR 11 on Chr 1**

*RELT* was only found to show association with MDV survival in Line WL5. In fact, Line WL5 only showed significant association with the *RELT* gene from all elements tested. P-values suggest one block in Line WL5, but complex blocks in other lines. The results do not allow dissection of the exact causative mutation.

**QTLR 13 on Chr 2**

*TRANK1* (Tetratricopeptide Repeat and ANKyrin Repeat Containing 1) was the only element tested in QTLR 13. Only 6 markers in Line WL1 were significant. P-values suggest a complex haplotype pattern, preventing localization of the putative quantitative cause.

**QTLR 19 on Chr 4**

The *CTNNA2* gene was the only element tested for MD association in this QTLR. Significant to highly significant effects were found in the white egg Lines WL3 and WL4, in the brown egg Lines WPR2 and RIR1, and across lines. In addition, the downstream gene, *CD8A*, was genotyped and gave indications of gene duplication and hence could not be genotyped, but thus a likely candidate QTG of the QTLR.

**QTLR 20 on Chr 5**

lncRNAs 6, 7 and 8 were tested within this QTLR. The white egg Line WL1 showed significance for both markers in lncRNA 6. The one marker in lncRNA 7 was not found to be significant, but the one marker tested from lncRNA 8 did show association with MDV survival. lncRNA 6 was not significant in the brown egg Line WPR1, but lncRNA 7 and one marker in lncRNA 8 were significant in this line. These results suggest two independent causative elements in this QTLR: one linked to lncRNA 6 distributed in Line WL1 and the other linked to lncRNA 7 in Line WPR1.

**QTLR 21 on Chr 5**

Three lncRNAs were tested within this QTLR, lncRNA 9, 10 and 11, and the results suggest more than one causative element in this region. Only lncRNA 10 was significant in the white egg Line WL2. One marker from each of the proximal and mid lncRNAs 9 and 10 was significant in the brown egg line WPR1, with 7 of the 15 markers in the distal lncRNA 11 being highly significant. Finally, 3 markers from distal lncRNA 11 were significant in the white egg line WL4. Thus, the putative distal quantitative trait element in this QTLR seems to be distributed in both white and brown egg lines.

**QTLR 29 on Chr 13**

The genes *HAVCR1* and *TIMD4* were tested in this QTLR (along with the functional mutation MD-32). HAVCR1 - Hepatitis A Virus Cellular Receptor 1 (T-Cell Immunoglobulin Mucin Receptor 1) is a receptor for TIMD4. Significant results were found for *HAVCR1* and *TIMD4* in the white egg Lines WL2 and WL3, and across lines. Distributions of P-values suggest complex haplotype blocks. Taking both these receptor/ligand genes together, along with the functional mutation in the gene ENSGALG00000004078, it would appear that there are at least 2 causal variants in QTLR 29: one distributed in Lines WL3 and WL4 in the region of *HAVCR1*, and one distributed in Line WL2 in the region of *TIMD4*.

**QTLR 30 on Chr 14**

Among the three elements tested in QTLR 30, only *SOCS1* showed some significance in one marker and the haplotypes in Line WL1, and the haplotypes only in Line WL4 (where one marker approached significance, P = 0.052). Thus, the most likely location of the causal element in this QTLR, is indeed in the region of *SOCS1*.

**QTLR 32 on Chr 16**

Both *TAP1* and *BG1* were found to be highly significant in Line WPR1 and across lines. Furthermore, the genes *BF1* and *BG2*, located either side of this QTLR, were also genotyped in this study, but both gave indications of gene duplication and hence could not be genotyped. The MHC genes are known to have gene duplications, multiple gene families, and repetitive regions, so this comes as no surprise. Since the MHC is known to have a large impact on MD resistance, it is tempting to attribute the quantitative effect found here to duplication of one or more genes, although further work is required to resolve this.

**QTLR 33 on Chr 17**

Some of the markers in *TLR4* were significant in Line WL3 and across lines. Complex haplotype patterns were clearly seen by the distribution of the P-values. Another candidate in this QTLR, BRINP1 (BMP/Retinoic Acid Inducible Neural Specific 1), inhibits cell proliferation by negative regulation of the G1/S transition. It is known to mediate non-classical cell death and regulates expression of components of the plasminogen pathway. No within-line test was significant, but two markers and the haplotypes were highly significant across lines. Taken together, the results from these two genes in QTLR 33 suggest *TLR4* as the most likely region of the QTG.

**QTLR 34 on Chr 18**

Marker CD7_697 was highly significant in Line WL2 and across lines. Line WL2 was tested only by this marker, and *CD7* was the only gene tested in this QTLR. Only 3 markers in this gene were tested, making it difficult to draw conclusions from these results.

**QTLR 36 on Chr 26**

*TREML2* shares QTLR 36 with the functional mutation MD-44 in ENSGALG00000003188 (Supplementary Table 7), which was significant in Line WL4. *TREML2* was significant only in Line WL3, but its most significant markers were very close to ENSGALG00000003188. Thus, the most likely location of the causal element for MDV resistance is upstream of *TREML2*.
