# Supplementary figures and images for: Mapping QTL Associated with Resistance to Avian Oncogenic Marek’s Disease Virus (MDV) Reveals Major Candidate Genes and Variants
Source: Genes (Basel). 2020 Aug 30;11(9):1019. doi: 10.3390/genes11091019 (PMC7564597; doi:10.3390/genes11091019)

## Slide 1
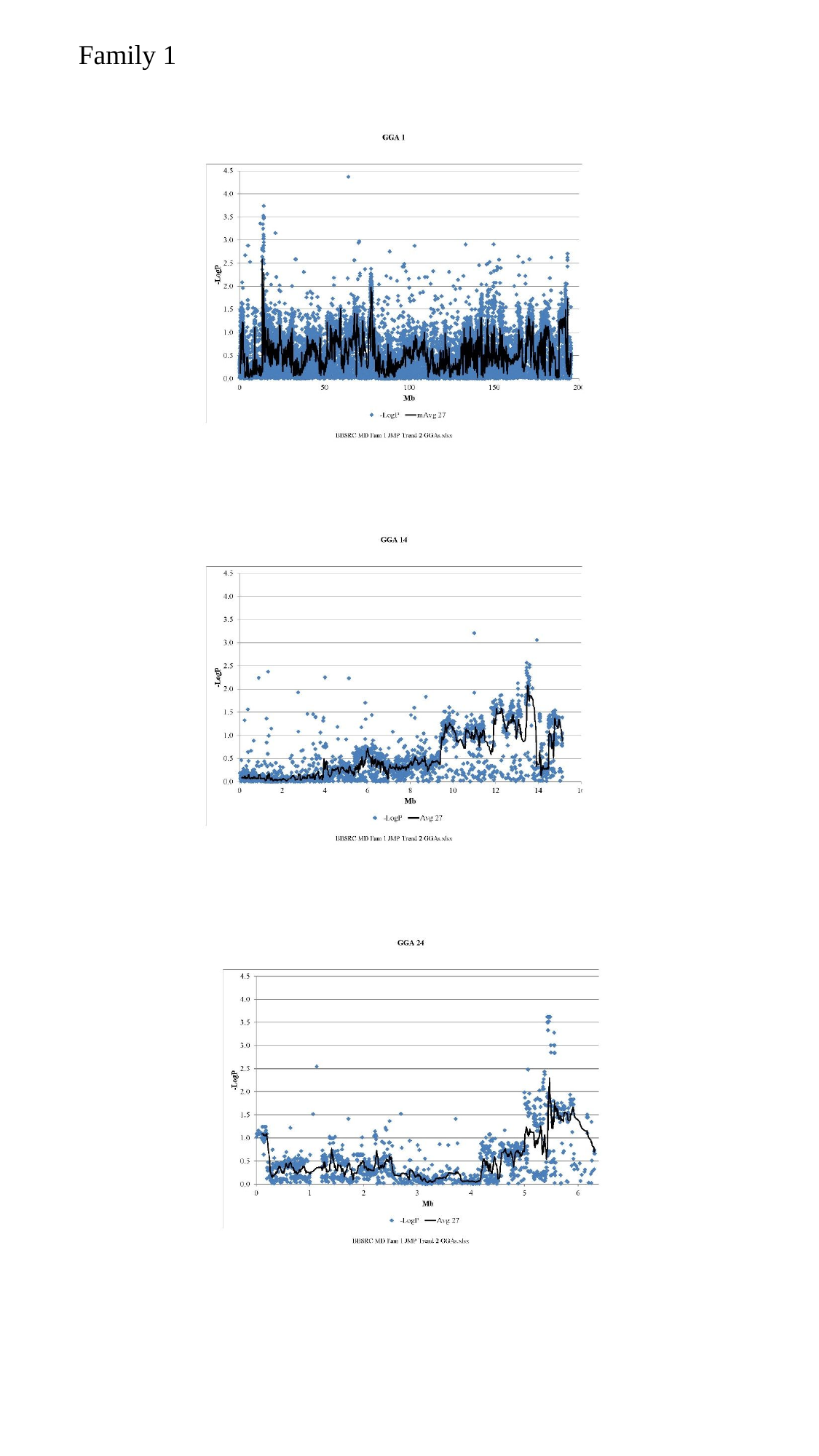

Family 1

## Slide 2
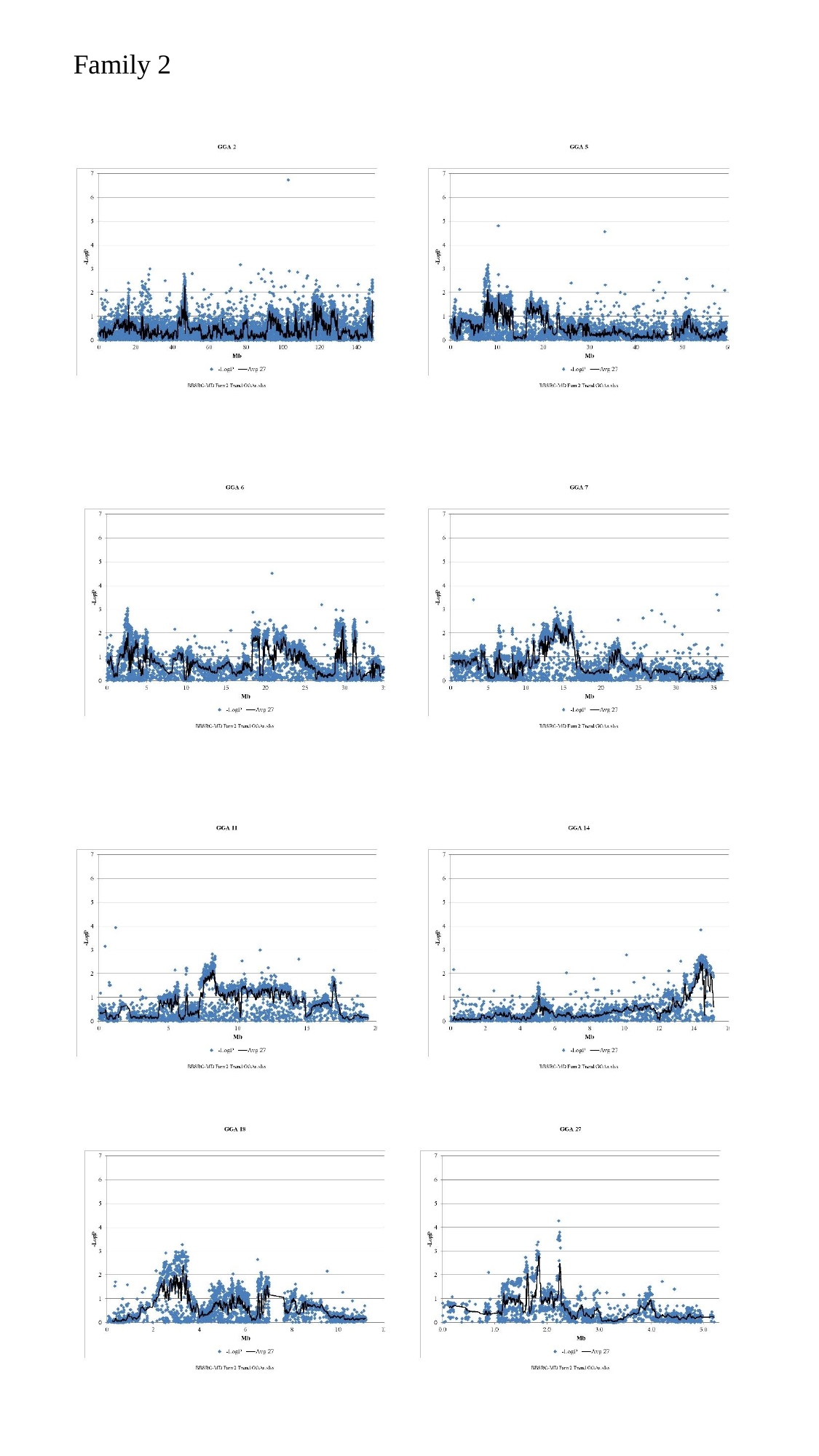

Family 2

## Slide 3
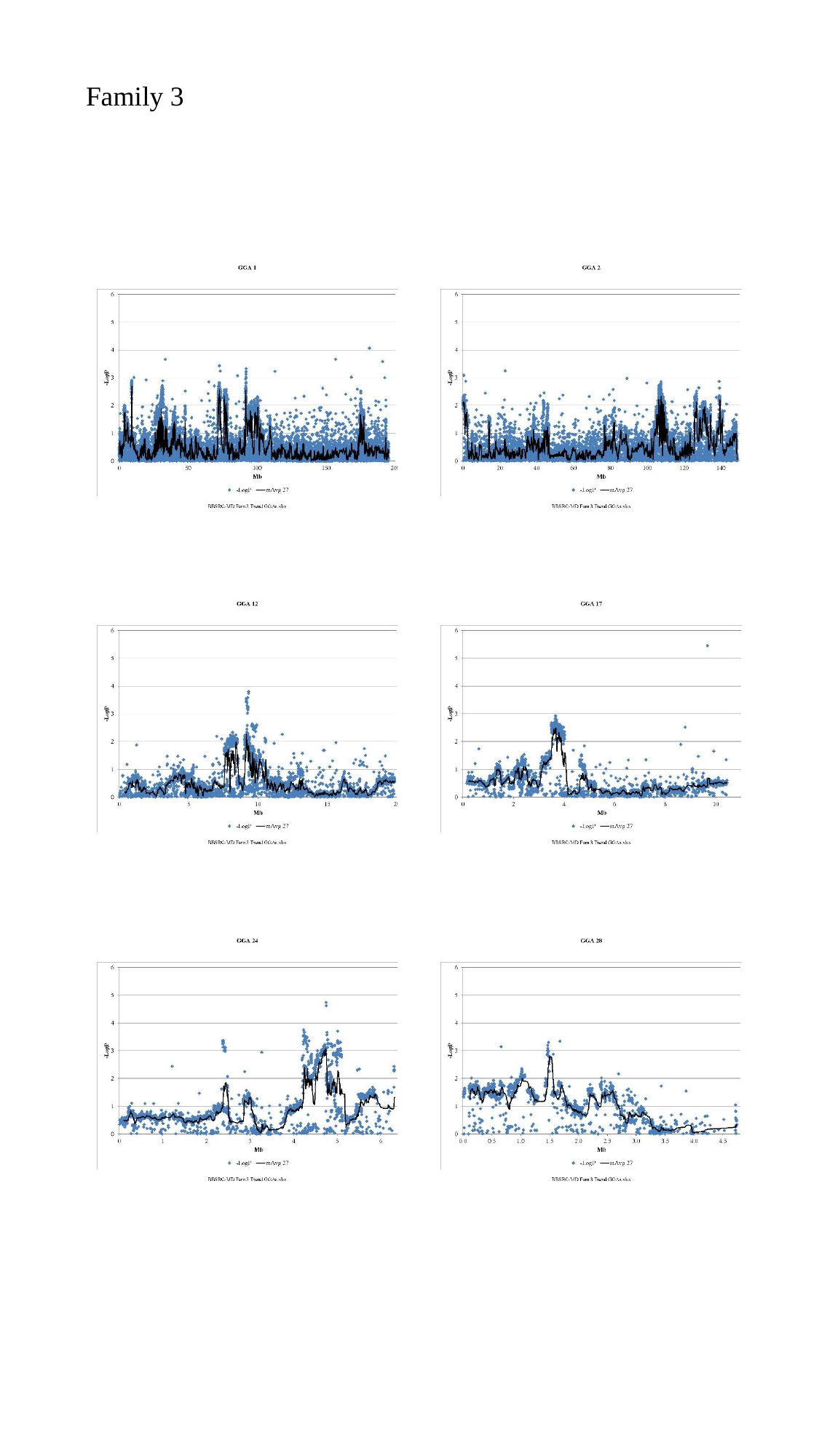

Family 3

## Slide 4
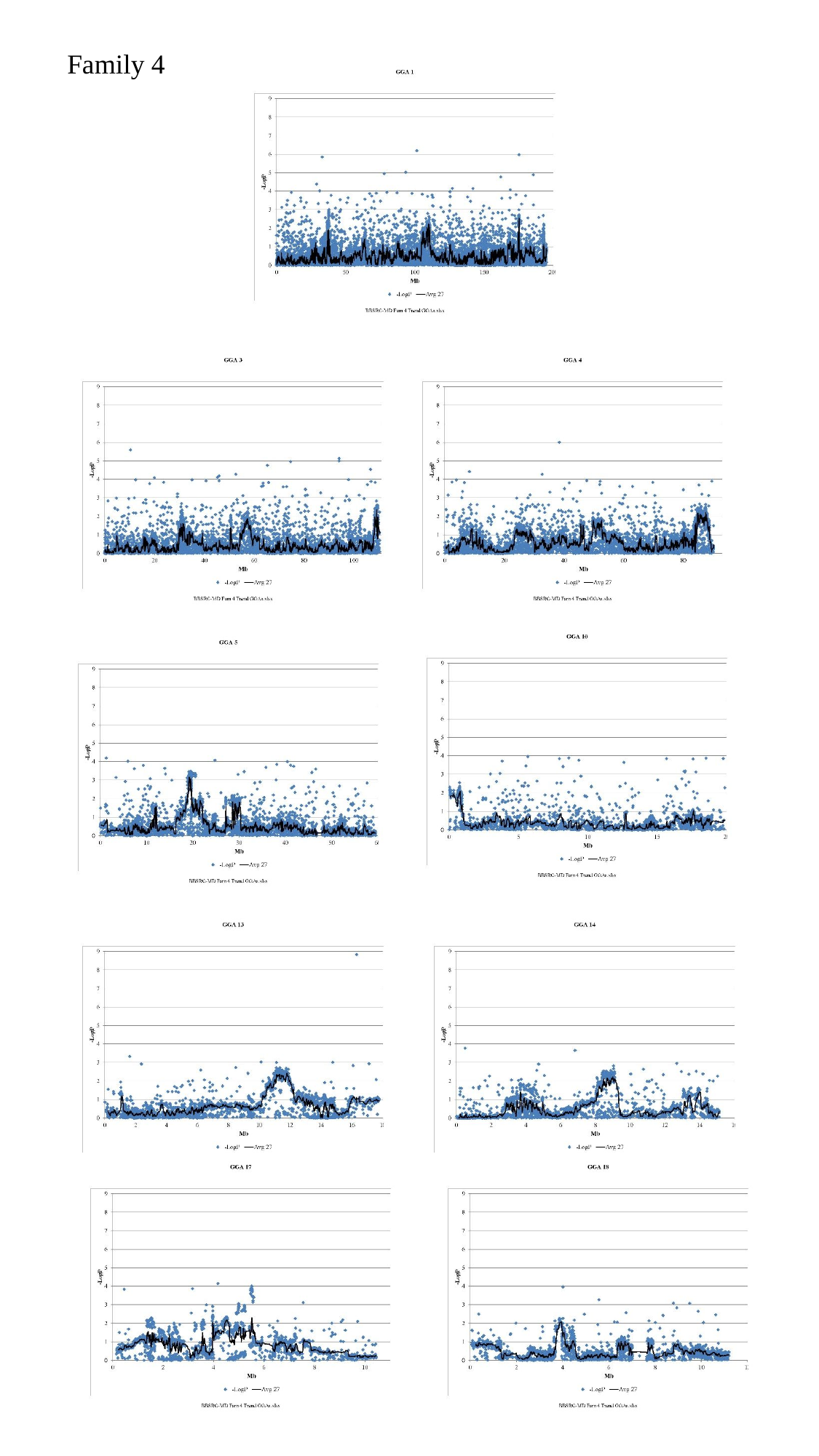

Family 4

## Slide 5
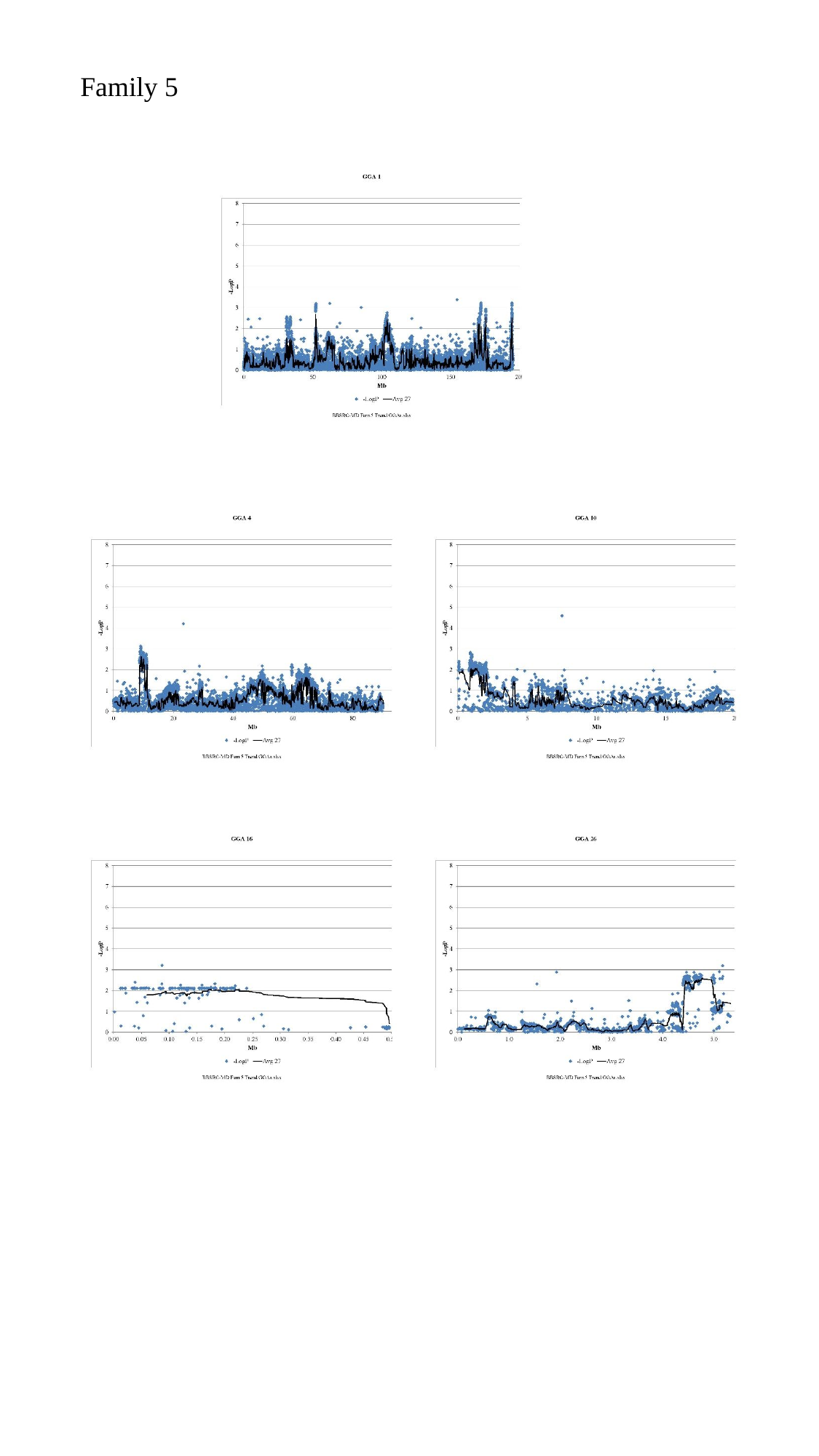

Family 5

Supplement: Supplementary file 1 [file genes-11-01019-s001.zip › S1 Figure.pptx]
